# Identification of Three Novel and One Known Mutation in the *WFS1* Gene in Four Unrelated Turkish Families: The Role of Homozygosity Mapping in the Early Diagnosis

**DOI:** 10.4274/jcrpe.galenos.2020.2020.0152

**Published:** 2021-02-26

**Authors:** Maha Sherif, Hüseyin Demirbilek, Atilla Çayır, Sophia Tahir, Büşra Çavdarlı, Meliha Demiral, Ayşe Nurcan Cebeci, Doğuş Vurallı, Sofia Asim Rahman, Edip Unal, Gönül Büyükyılmaz, Riza Taner Baran, Mehmet Nuri Özbek, Khalid Hussain

**Affiliations:** 1 University College London, Institute of Child Health, Developmental Endocrinology Research Group, Clinical and Molecular Genetics Unit, London, United Kingdom; 2 Diyarbakır Children’s Hospital, Clinic of Paediatric Endocrinology, Diyarbakır, Turkey; 3 Hacettepe University Faculty of Medicine, Department of Pediatric Endocrinology, Ankara, Turkey; 4 Regional Training and Research Hospital, Clinic of Paediatric Endocrinology, Erzurum, Turkey; 5 Ankara City Hospital, Clinic of Medical Genetics, Ankara, Turkey; 6 Gazi Yaşargil Training and Research Hospital, Clinic of Pediatric Endocrinology, Diyarbakır, Turkey; 7 Derince Training and Research Hospital, Clinic of Paediatric Endocrinology, Kocaeli, Turkey; 8 Ankara City Hospital, Clinic of Pediatric Endocrinology, Ankara, Turkey; 9 Sidra Medicine, Department of Pediatrics, Division of Endocrinology, Doha, Qatar

**Keywords:** Wolfram syndrome, WFS1, diabetes mellitus, diabetes insipidus, optic atrophy, sensorineural deafness

## Abstract

**Objective:**

Bi-allelic mutations in the *wolframin* gene (*WFS1*) cause Wolfram syndrome 1 (WS1 or DIDMOAD) characterized by non-autoimmune diabetes mellitus, optic atrophy, diabetes insipidus, sensorineural deafness, urinary tract abnormalities, and neuropsychiatric disorders. Patients presenting with an incomplete phenotype of WS1 were evaluated using homozygosity mapping and subsequent whole-exome sequencing.

**Methods:**

Four unrelated consanguineous Turkish families, including seven affected children, and their unaffected parents and siblings were evaluated. Homozygosity mapping was performed, followed by whole-exome sequencing of *WFS1*. Mutations were classified according to results of “*in silico*” analyses, protein prediction, and functional consequences.

**Results:**

Homozygosity mapping confirmed shared homozygous regions on chromosome 4 (chr4p16.1) between the affected individuals, that was absent in their unaffected siblings. Exome sequencing identified three novel (c.1215T>A, c.554G>A, c.1525_1540dup) and one known (c.1522_1523delTA) mutations in *WFS1*. All mutations were predicted to cause stop codon leading to early termination of protein synthesis and complete loss-of-function. All patients were found to be homozygous for the change, with parents and other unaffected siblings being carriers.

**Conclusion:**

Our study expands the mutation spectrum of *WSF1* mutations with three novel mutations. Homozygosity mapping may provide enrichment for molecular genetic analysis and early diagnosis of WS1 patients with incomplete phenotype, particularly in consanguineous pedigrees.

**What is already known on this topic?**

Wolfram syndrome 1 (WS1) is a clinically heterogeneous disease with variable manifestations and progression pattern depending on the underlying molecular genetic aetiology. Patients may present with incomplete phenotype, but the disease has a progressive nature with a negative impact of poor glycaemic control. Identification of molecular genetic aetiology provides early diagnostic confirmation and thereby an opportunity to detect and manage the subtle symptoms more appropriately.

**What this study adds?**

Our study expands the mutation database of WFS1 with three novel variants and provides further insights into the genotype and phenotype association. We used homozygosity mapping as an adjunctive tool, which contributed to early detection of molecular genetic etiology in cases that presented with incomplete WS1 phenotype.

## Introduction

Wolfram syndrome (WS), first described in 1938 by Wolfram and Wagener (1) in four siblings, is an autosomal recessive disorder characterized by early-onset diabetes mellitus (DM), progressive neurodegeneration, endocrine dysfunction, and psychiatric disorders (2). WS1 is also known as a syndrome with the acronym DIDMOAD, which describes the frequent clinical features of the disease; diabetes insipidus (DI) and DM with optic atrophy (OA) and deafness. As WS1 is a progressive degenerative disease, additional clinical features including ataxia, urinary tract and renal disorders, and psychiatric disorders may present later in life (2).

WS1 is a rare cause of early-onset, non-autoimmune DM, which is the most common clinical feature, and usually occurs within the first decade of life (median age: 6 years). This is followed by progressive OA (median age of presentation at around 11 years), which first begins with colour and peripheral vision loss and can eventually lead to blindness over the next decade of life, as neurodegeneration progresses (3). All four clinical features described by DIDMOAD were observed in around 66% of patients in a review of 392 WS patients (4).

Biallelic loss-of-function mutations in wolframin endoplasmic reticulum (ER) transmembrane glycoprotein gene (*WFS1*), located at chromosomal position 4p16.1, accounts for the molecular genetic aetiology of WS1 (5). *WFS1* encodes for the protein wolframin, which is expressed ubiquitously, while the steady-state levels vary significantly among organs (6). It is highly expressed in brain neurons, pancreas, heart and muscle, and lower expression is observed within the liver and skeletal muscle, while the lowest expression is in kidney and spleen (6). Within the pancreas, wolframin has a higher expression in the islet cells than the pancreatic exocrine cells (5). Wolframin, a transmembrane glycoprotein composed of a cytoplasmic N-terminal domain, a central nine-transmembrane domain, and a luminal C-terminus, is predominantly localized in the ER (7). It is involved in the regulation of ER-stress, which is critically important in establishing intracellular homeostasis, integrity and survival of the cell (8,9). Wolframin is primarily involved in the unfolded protein response (UPR), which transduces the stimulus for increased unfolded proteins and maintains the balance between anti-apoptotic and pro-apoptotic processes (9,10). The UPR regulates ER-stress by eliminating misfolded proteins or attenuating protein translation (11,12). Loss-of-function in wolframin causes decreased UPR activity, and thereby a chronic ER-stress mediated apoptosis and cell death (8,9). This eventually causes both neurodegeneration and loss of beta-cell mass (13). Wolframin also plays an essential role in the stimulus-response coupling mechanism, which regulates beta-cell insulin synthesis and secretion (13,14).

WS1 is a clinically heterogeneous disease with variable presentation as well as progression pattern depending on the underlying molecular genetic aetiology (4,15,16). Such diversity can make diagnosing WS1 difficult, especially in the context of an outbred population, multiple genes may need to be explored and sequenced. However, in consanguineous families, homozygosity mapping can prove to be an efficient tool to localize causative genes for recessive traits. It allows targeting of specific chromosomal regions of DNA that are shared only by affected individuals, thereby facilitating the process of finding candidate genes and detecting mutations, when used alongside exome sequencing.

In the present study, evaluation was performed of seven affected individuals and their apparently healthy relatives from four unrelated, consanguineous Turkish families with a variable clinical phenotype which was found to be due to three novel and one previously described *WFS1* mutation.

## Methods

### Patients

In this study, seven affected patients (six males and one female) were evaluated who had presented with rare forms of DM and sensorineural deafness (SND) together with their apparently healthy parents and siblings so that a total of 21 individuals from four unrelated families were included. All patients are from first-degree consanguineous parents. Homozygosity mapping was performed in all patients and their unaffected siblings, from all families. Specific chromosomal regions were identified which were shared by the patients alone, and absent in their unaffected siblings. In these regions candidate genes were then identified, and exome sequencing data was used to detect mutations in the region of interest.

### Family 1

**Figure 1:**
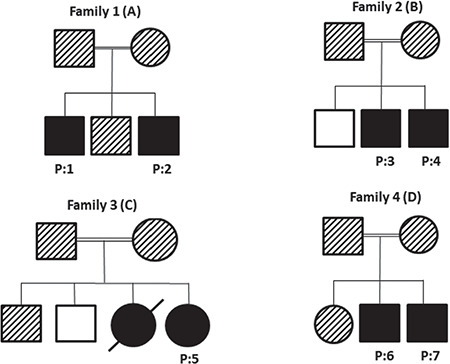
A, B, C, D) Family pedigrees of Wolfram syndrome patients. Black-filled boxes refer to homozygous and clinically affected members, while black shaded boxes indicate heterozygosity and completely empty boxes refer to mutation-negative unaffected family members (A female sibling of P5 did not undergo mutation analysis, but she had clinical features of WS1 similar to P5. It was therefore thought that she presumably had an identical mutation and is displayed with black-filled box)

Family 1 consists of three male children, two affected with DM and SND, and one unaffected. Parents were first cousins (Figure 1A).

**Patient 1** was born at term with a birth weight of 2.6 kg. At the age of seven years, he presented with polyuria and polydipsia. Blood glucose level was 340 mg/dL (18.9 mmol/L) at the time of diagnosis with no diabetic ketoacidosis (DKA). Diabetes autoantibodies [islet cell antibodies (ICA), insulin autoantibodies (IAA), and GAD65] were negative. He was started on insulin therapy. He also developed SND when he was about 13 years old. A DNA sample was collected due to non-autoimmune, early-onset DM and SND. Although he had a history of decreased visual acuity observed at the age of eight years, the diagnosis of OA was considered only after reassessment due to genetically proven WS1 diagnosis (Table 1). During the follow-up, he developed all clinical features of WS1, as displayed in Table 1.

**Patient 2** was born at term with a birth weight of 2.8 kg. At the age of 11 years, he presented with polyuria and polydipsia. Blood glucose level was 301 mg/dL (16.7 mmol/L) with no DKA at the time of diagnosis. Diabetes autoantibodies (ICA, IAA, and GAD65) were negative. He also had a history of decreased visual acuity, which was first observed at the age of eight years. Blood sample for DNA was collected due to his early onset, non-autoimmune diabetes and medical history of his elder brother. A diagnosis of OA was also considered when a full ophthalmological evaluation was performed after the results of DNA analysis. These two patients from family 1, unfortunately, did not attend their regular follow-up visits. At the latest follow-up visit, when he was 16 years old, his audiological evaluation also revealed the diagnosis of SND, although the patient was not suffering from a hearing problem (Figure 1A). The clinical features of WS1 and the age of onset for symptoms in this patient are displayed in Table 1.

### Family 2

Family 2 is a large consanguineous family with two affected male siblings (Figure 1B).** Patient 3 (P3)** was born at term with a birth weight of 2.9 kg. At the age of two years, he was diagnosed with DM. At presentation, his blood glucose level was 270 mg/dL (15 mmol/L), and diabetes autoantibodies (ICA, IAA, and GAD65) were negative. He was started on insulin therapy. He also had a history of SND, which was noticed at the age of two years. He had no other features of WS1 at the time of the DNA sampling (at the age of 17 years), while he developed central DI at the age of 20 years. He also had mild-moderate mental retardation and emotional instability (Table 1).

**Patient 4 (P4) **was born at term with a birth weight of 2.7 kg. At the age of five years, he was diagnosed with DM. The blood glucose level at admission was 306 mg/dL (17 mmol/L), and diabetes autoantibodies (ICA, IAA, and GAD65) were negative. Insulin therapy was commenced. He also had a history of SND detected at the age of two years. At his latest follow-up visit at the age of 20 years he had no other feature of WS1, but a mild developmental delay was observed (Table 1).

### Family 3

**Patient 5 (P5)** is a female patient born to first-cousin parents (Figure 1C). She had two unaffected male siblings and a history of one sister and one first-cousin with DM and SND who both died with unknown aetiology. She was born at term with a birth weight of 2.5 kg. She had SND, which was first noticed at the age of two years and required hearing aid at the age of five years-old. She presented with polyuria and polydipsia at the age of six years. At presentation, she had a blood glucose level of 360 mg/dL (20 mmol/L) with no DKA. The diabetes autoantibodies (ICA, IAA, and GAD65), were negative. A diagnosis of DM was considered, and insulin therapy commenced. She developed decreased visual acuity, which was first noticed at the age of 10 years, and a diagnosis of OA was considered at the age of 13 years. She developed central DI at the age of 16 and had all the cardinal features of WS1 at her latest follow-up visit at 18 years-old although there were no renal and psychiatric disorders (Table 1).

### Family 4

Family 4 is a first-degree consanguineous family with two affected male (patients 5 and 6) and one unaffected female sibling (Figure 1D).

**Patient 6 (P6)** was born at term with a birth weight of 2.7 kg. At the age of five years, he presented with polyuria and polydipsia. His fasting blood glucose level was 340 mg/dL (18.9 mmol/L), and diabetes autoantibodies (ICA, IAA, and GAD65) were negative. A diagnosis of DM was considered, and insulin therapy commenced. He subsequently developed visual and hearing deficits around the age of seven years. His developmental milestones were achieved appropriately for age. The younger brother (P7), was born at term with a birth weight of 2.9 kg. At the age of six years, he presented with polyuria and polydipsia, and a fasting blood glucose level of 234 mg/dL (13 mmol/L). Diabetes autoantibodies (ICA, IAA, and GAD65) were also negative. He had no visual or hearing loss at the time of this study (Table 1).

The ethical approval was granted by University College of London (UCL), Institute of Child Health, Great Ormond Street Hospital for Children (R&D number: 12CM47). Informed consent was obtained from all patients or their legal guardians and unaffected family members.

### Molecular Genetics Analysis

Families were originally recruited alongside a cohort of other families in a study to identify rare causes of DM and SND. Not all patients had evidence of OA at the time of the presentation, and therefore WS1 was not considered initially. Genomic DNA was isolated through standard techniques at the UCL Genomics centre. DNA samples from these patients were sent for homozygosity mapping at the UCL Genomics centre, as all patients belonged to consanguineous parents. The Illumina microarray platform was used for genotyping, following the Infinium HD Ultra Assay protocol (Rev B, 2010, Illumina Inc, San Diego, USA). Results were generated using the Illumina Genomestudio software, and copy number variation and loss of heterozygosity data was generated (cnvPartition v3.1.6, Illumina). The minimum homozygous region size was 1 Mb, with a minimum of 50 consecutive SNPs. Further, to identify rare variants possibly explaining early-onset DM and SND, exome sequencing was performed out of UCL. Primer 3 software was used to design the primers for the *WFS1* gene. The sequencing reaction was conducted using the BigDye Terminator V1.1 Cycle Sequencing kit (Applied BioSystems, Foster City, CA, USA). The sequences were compared to a reference sequence using the Sequencher® 5.3 software. The variants were classified based on the 2015 American College of Medical Genetics and Genomics and Association for Molecular Pathology guidelines using InterVar (17). The variants were also classified concerning effects on protein synthesis, genotypic classification and functional consequences using the classifications described by de Heredia et al (4) and Rohayem et al (16) (Table 2).

## Results

**Figure 2:**
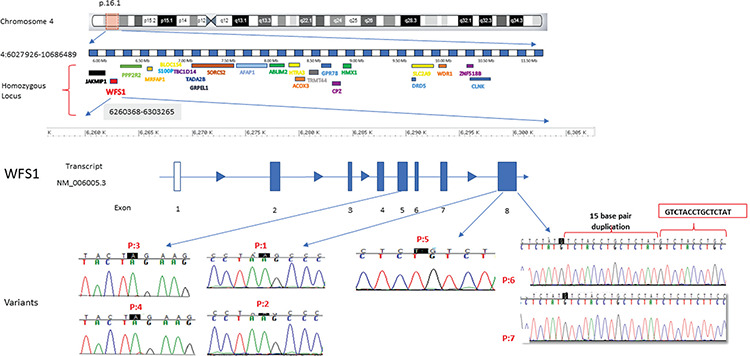
Homozygosity mapping showed a shared region between affected members where the *WFS1* gene was located in the same region. Electropherogram of the WFS1 analysis for patients 1-7

Homozygosity mapping results demonstrated shared homozygous regions on chromosome 4 (chr4p16.1) between the affected individuals, and these were absent from their unaffected siblings. The search was targeted in this region. The 4p16.1 locus contains 81 genes of which 51 are protein-coding. Although 23 of these genes are defined in the Online Mendelian Inheritance in Man database (OMIM), only four genes (*WFS1, HMX1, SLC2A9, DRD5*) are associated with a phenotype in the OMIM morbid list (Figure 2). We performed whole-exome sequencing that identified *WFS1* gene mutations, which was confirmed by Sanger sequencing (Table 2). In total, we analyzed samples from 21 individuals, including affected subjects and their apparently unaffected family members.

A novel nonsense c.1215T>A (p.Tyr405Ter) variant was detected in exon 8 of the *WFS1* gene in family 1 (P1 and P2) (Table 2). This variant has not been listed in mutation databases (HGMD, Clinvar), sequence variant databases (Exome Variant Server, dbSNP, EXAC and 1000genome) or not published elsewhere in the literature search including Google and PubMed databases. The pathogenicity and classification of the variant according to various classifications are displayed in Table 2.

In family 2 (P3 and P4), we identified another novel nonsense variant in exon 5 of *WFS1*, where the base pair change c.554G>A, leads to early termination of the protein chain (p.Trp185Ter), leading to synthesis of a truncated protein (Table 2). The pathogenicity and classification of the variant according to various classifications are displayed in Table 2. We first presented this mutation at European Society For Paediatric Endocrinology 2014 meeting (18), but shortly after our report, another group published the same variant in a Jordanian family (19).

In family 3 (P5), a known frameshift/nonsense mutation (c.1522-1523delTA, p.Y508X) was identified in the *WFS1 *gene. This mutation has been reported only once before, in 2006, in two Turkish male siblings with features of WS1 and suicidal behaviour (20). The pathogenicity and classification of the variant according to various classifications are displayed in Table 2.

In family 4 (P6 and P7), a novel C.1525_1540dup15 duplication mutation was identified. This variant has not been listed in mutation databases (HGMD, Clinvar), sequence variant databases (Exome Variant Server, dbSNP, EXAC and 1000 genome) and not published elsewhere in the literature search including Google and PubMed databases. The pathogenicity and classification of the variant according to various classifications are displayed in Table 2.

## Discussion

In the present study, we evaluated the clinical characteristics, underlying molecular genetics and follow-up of seven patients with WS and their 14 unaffected relatives from four unrelated, consanguineous Turkish families. As the patients’ phenotypes were incomplete for WS1 diagnosis, homozygosity mapping was used to enrich the molecular genetic analysis and identified three novel variants, and one previously reported mutation in another Turkish family from the same geographical location (20).

*WFS1* gene is intolerant to the loss-of-function mutations - the score of probability for being loss-of-function intolerant is 1.0 (21). Functional studies and protein analysis of fibroblast cell lines of WS1 patients have shown that nonsense, splicing site and frameshift mutations of *WFS1* cause nonsense transcripts that are unstable *in vivo* and rapidly degraded by nonsense-mediated mRNA decay (6,22,23). Missense variants have been shown to cause a WS1 phenotype by affecting post-transcriptional modifications, protein stability and regulation of the degradation of wolframin transcripts (12). Besides, missense mutations are predicted to cause reduced half-time and low steady-state level of the wolframin, and thus are suggested to have a dosage-sensitive effect (6,22,23).

To date more than 330 variants have been described in the *WFS1* gene (The Human Gene Mutation Database website: http://www.hgmd.cf.ac.uk/ac/index.php, latest access 22^nd ^May 2020). Of these around 230 have been reported to be associated with a WS1 phenotype. The common type of mutations include missense, nonsense, frameshift, splice-site mutations, in-frame deletions/insertions or duplications. The majority of *WFS1* mutations have been detected in exon-8, which accounts for about 86% of variants detected (24). Mutations detected in our case series were nonsense (n=2), deletion (n=1) and duplication (n=1) mutations leading to a stop codon, thereby, early termination of the protein wolframin.

**Figure 3:**
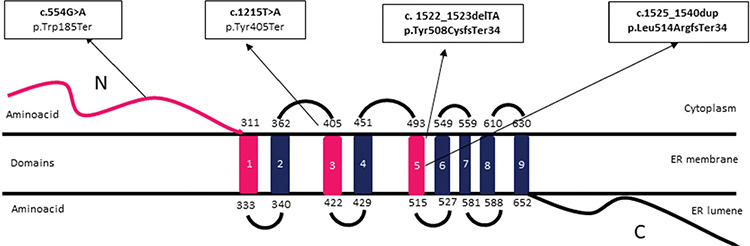
A motif of *WFS1* showing the NH2-terminal, nine transmembrane and the -COOH terminal domains and location of mutations detected in the present report

The novel nonsense c.1215T>A (p.Tyr405Ter) variant is located on the third transmembrane domain of the *WFS1* gene (Figure 3), and results in a premature stop codon and early termination of the protein chain (PVS1) (Table 2). This nonsense mutation is predicted to lead to nonsense-mediated mRNA decay and complete depletion of wolframin protein, and therefore complete loss-of-function. Homozygous affected members and heterozygous/wildtype variants in the unaffected family members showed a phenotype and genotype co-segregation.

The novel nonsense c.554G>A (p.Trp185Ter) variant leads to early termination of the protein chain and thereby synthesis of a truncated protein (Table 2). This is an “N terminal” stop-gain variant that causes complete loss-of-function due to premature termination of protein synthesis and rapid degradation of the truncated transcripts (Table 2 and Figure 3). Although this mutation is predicted to severely affect wolframin expression leading to complete loss-of-function, the clinical phenotype in our cases was relatively mild compared to patients from the Jordanian family (19). The age of onset for DM was similar and between 2-5 years-old in both families. However, in our patients SND was observed earlier (two years vs five years). The most striking discrepancy was the development of OA and DI, which were detected around the ages of four years (DI) and five years (OA) in Jordanian patients. However, none of our cases developed OA until their latest follow-up visit at the age of 20 and 22 years. None of our cases developed urinary tract abnormalities which were observed in one of the Jordanian patients (hydronephrosis and gall bladder stones). Patients from both families had a moderate intellectual disability.

The previously published frameshift/nonsense, c.1522-1523delTA (p.Y508X) mutation causes deletion of two base-pairs (TA) in exon-8 of *WFS1 (*Figure 3). It causes disturbance in the normal reading frame which result in early termination of the amino acid sequence and synthesis of a truncated protein. This mutation has been reported only once before, in 2006, in two Turkish male siblings with WS1 features and suicidal behaviour (20). The previously reported two siblings were from the same city as our case, while both families were not related. The age of DM in the first report and our case were similar, while our case developed hearing loss at an earlier age of two years, suggesting congenital SND. Besides, our case developed other clinical features at a later age compared to the first cases and still has not yet developed psychiatric complications nor urinary tract problems.

The novel C.1525_1540dup15 mutation duplicates 15-base pairs in the five amino acids from V509_Y513 (Figure 3). This mutation causes production of a stop codon and changes the reading frame and thereby termination of protein synthesis at the 34^th^ amino acid in the sequence. Sanger sequencing confirmed that two affected siblings had a homozygous mutation, whereas their unaffected family members were heterozygote carriers.

WS1 is a clinically heterogeneous disorder with variable age of onset for clinical features. DM and OA are the most common presenting features with a frequency of 98.2% and 82.1%, respectively (4). The disease has a progressive nature, and other components of DIDMOAD develop within a variable timeframe depending on mutation characteristics. Therefore, mutation analysis of the *WFS1* gene in patients with any two DIDMOAD symptoms is warranted for early detection of WS1 patients (4,15,16). In our case series, the age of onset for DM was similar to previous reports and consistent with the mutations characteristics, while OA was detected at a lower rate and with variable timing. Besides, at the time of the genetic analysis, OA was not confirmed in the majority of cases (only two out of seven). SND was the second most prominent feature, which was detected in five out of seven patients at the time of the genetic analysis. Therefore homozygosity mapping was performed, which provides enrichment for detection of mutations in *WFS1*. Homozygosity mapping has previously been reported as contributing to the detection of novel mutations, as well as a new coding region responsible from the WS phenotype (25). Mutations in this second locus cause a distinct WS phenotype with upper gastrointestinal tract bleeding and the absence of DI (WS2) (25).

Age of presentation for deafness can be quite variable ranging from severe congenital deafness to late-onset mild and progressive hearing loss (4,26). The age of presentation in three out of six patients who developed SND was two years. Heterozygous mutations in *WFS1* are also associated with a dominant form of hearing loss, known as low-frequency sensorineural hearing loss (27). Although we were not able to perform audiological evaluation for family members who were carrying a heterozygous mutation, none had declared symptoms of hearing loss. Nevertheless, diagnostic evaluation based on parents or patients’ declaration or examination without using standardized method may result in underdiagnosis of hearing loss, which suggests a need for using standardized audiological methods for the evaluation of patients presenting with WS1 symptoms (26).

Urinary tract abnormalities, including upper urinary tract dilatation (hydroureteronephrosis), recurrent urinary tract infections, urinary incontinence due to atonic bladder, and end-stage renal failure are common features of WS1 (2,28). The rate of urinary system problems has been reported in up to 90% with a median age of 20 years and three peaks observed at the age of 13, 21 and 33 years (2). Indeed, two patients with the c.554G>A and c. 1522-1523delTA mutations have been previously reported to have urinary tract problems (19,20). Nevertheless, until their latest follow-up visits at the age of 22 years, none of our patients with identical mutations developed urinary tract problem.

Mutations that cause earlier presentation of DM are suggested to also cause accelerated neurodegeneration (4,16). Besides, glucotoxicity is also associated with an increased risk of neurodegenerative disorders in WS1 patients (16). Good glycaemic control (HbA1c<7.5%) has been shown to correlate with a lower rate of DI, deafness, and neurological and psychiatric symptoms (16). In our case series, early-onset DM and SND were seen in the majority of cases. However, other neurological features of WS1 developed later over wide range of ages. Notably, two patients with the earliest presentation for DM and SND had not developed OA at the ages of 20 and 22 years. One of these patients had also not yet developed other features of WS1 except for DM and SND, while the other developed DM, DI and SND in addition to psychiatric symptoms (Table 1).

WS1 patients present with a wide variety of clinical symptoms and signs due to the clinically heterogenous nature of the disease. Furthermore, the varying ages of presentation of symptoms, and the different rates of progression, makes WS1 a very difficult syndrome to diagnose in the early stages, especially when only one symptom may be apparent, consequently leading to delay in the treatment and management. With the use of homozygosity mapping, we were able to narrow down specific regions of the human genome, which were shared only by affected individuals of different families, allowing us to fast track the search for the causative gene.

### Study Limitations

A limitation of the present study was the unavailability of functional analyses of the novel variants identified in our cases.

## Conclusion

In conclusion, our study expands the mutation spectrum of WSF1 with three novel nonsense variants in three unrelated consanguineous families, confirming variable phenotypical expression and heterogeneity in presenting features as well as the progressive nature of the disease. The prominent WS1 features in our cases were early onset SND, lower rate and delay in development of OA, and lack of urinary tract problems. Homozygosity mapping proved to be a useful tool for enrichment of molecular genetic analysis in the early diagnosis of WS1 patients with an incomplete phenotype, particularly those with no OA, or belonging to consanguineous pedigrees.

## Figures and Tables

**Table 1:**
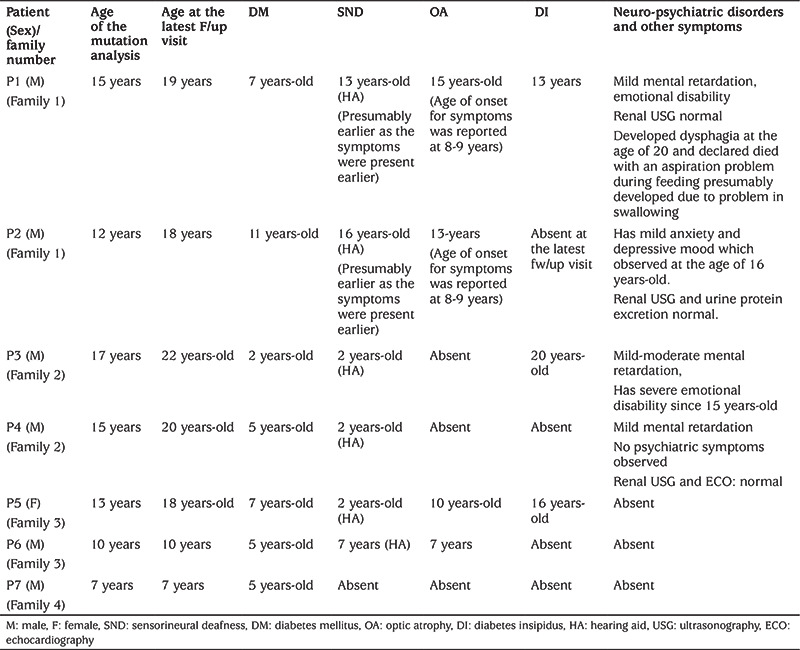
Age of onset for clinical characteristics for the WS1 features in seven cases with homozygous *WFS1* mutations

**Table 2:**
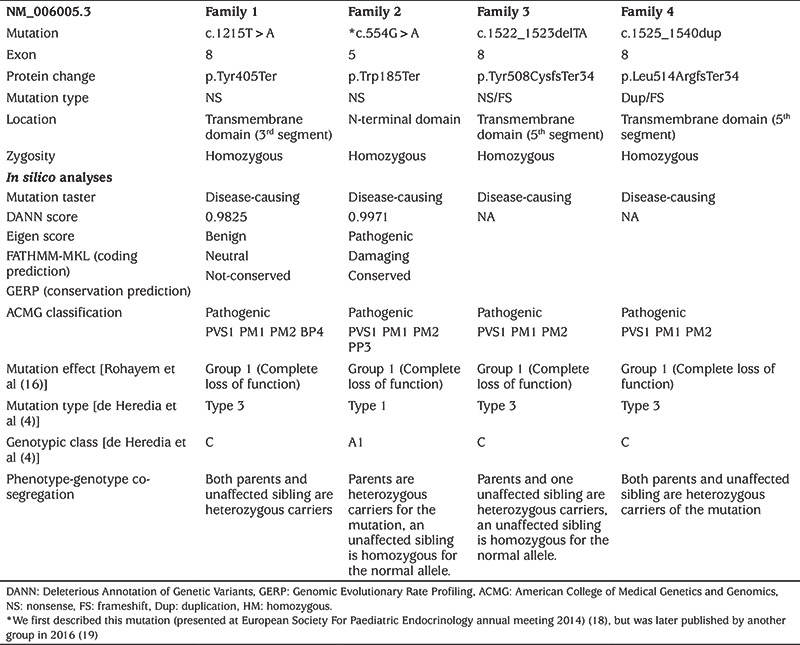
Mutations characteristics, results of *“in silico”* analyses, protein prediction, functional consequences and individuals with particular mutations
